# SARS-COV-2 Infection and Kawasaki Disease: Case Report of a Hitherto Unrecognized Association

**DOI:** 10.3389/fped.2020.00398

**Published:** 2020-07-03

**Authors:** Marco Cazzaniga, Lucia Augusta Baselli, Rolando Cimaz, Sophie Suzanne Guez, Raffaella Pinzani, Rosa Maria Dellepiane

**Affiliations:** ^1^Pediatric Intermediate Care Unit, Fondazione IRCCS Ca' Granda Ospedale Maggiore Policlinico, University of Milan, Milan, Italy; ^2^Pediatric Intermediate Care Unit, Fondazione IRCCS Ca' Granda Ospedale Maggiore Policlinico, Milan, Italy; ^3^Department of Clinical Sciences and Community Health, Research Center for Adult and Pediatric Rheumatic Diseases, University of Milan, Milan, Italy; ^4^Pediatric Highly Intensive Care Unit, Fondazione IRCCS Ca' Granda Ospedale Maggiore Policlinico, Milan, Italy

**Keywords:** Kawasaki disease, SARS-COV-2, children, paralytic ileus, cytokine storm

## Abstract

Coronavirus-associated disease (COVID-19) was firstly reported at the end of 2019. Generally, COVID-19 seems to be a less severe disease in children than in adults. According to the current literature, children account approximately for 2% of diagnosed COVID-19 cases. Northern Italy is one of the geographical areas mainly affected by the ongoing COVID-19 pandemic. We describe a pediatric patient diagnosed and treated for atypical/incomplete Kawasaki Disease (KD) complicated with paralytic ileus, who also resulted positive for SARS-COV-2.

## Introduction

Covid-19 infection is currently the most important health problem worldwide. According to the current literature, children account approximately for 2% of diagnosed COVID-19 cases (1–3). Even if most of medical efforts are aimed to fight this disease, physicians still have to care for other acute and chronic disorders. Kawasaki disease is not an exception, and there may be a risk that patients are not recognized early since patients may not seek medical care as usual for fear of getting infected (4). The association of Kawasaki disease and COVID-19 infection has to our knowledge been reported only once (5), we report a second case from Italy, currently the third most affected country in the world with regard to number of proven SARS-COV-2 infections.

## Case Report

We report the case of a 6-year-old, full term, previously healthy boy. In March 2020 he was seen by his pediatrician for fever lasting for 3 days, sore throat and asthenia, and antibiotic therapy with amoxicillin + clavulanic acid was prescribed. High fever persisted with vomiting and diarrhea appearing on day 4 and 5. On day 6 from onset of fever, erythematous rash in the back and hands, labial and conjunctival hyperemia appeared, and the child was admitted to a regional hospital. Laboratory tests showed white blood cells count of 10.300/mm^3^ (neutrophils 88.6%, lymphocytes 7.1%), Hb 11.3 g/dl, platelets 1,49,000/mm^3^, elevated liver enzymes (AST 73 U/L, ALT 189 U/L, GGT 128 U/L), C-reactive protein 13 mg/dL (nv < 5), fibrinogen (524 mg/dl, nv 165–350), ferritin (612 μg/L, nv 30–400), procalcitonin (5.05 μg/L, nv 0.02–0.06), hypoalbuminemia (2.7 g/dL), and hyponatremia (124 mEq/L). The nasopharyngeal respiratory pathogen testing by reverse transcription polymerase chain reaction test (RT-PCR) was positive for Rhinovirus and Enterovirus, and a chest X-ray was normal. Intravenous antibiotic therapy with cefotaxime and correction of hyponatremia was started. Given the COVID-19 pandemic period, the child was tested for SARS-COV-2 with nasopharyngeal swab, negative in two determinations. As the patient experienced worsening abdominal tension and pain, abdominal ultrasound and CT scan were performed with evidence of fluid in pelvis and right iliac fossa, spleen size at the upper limits, and no air-fluid levels. Plain abdominal X-ray showed gastrectasia and increased gas in the ileal loops. On day 7 of fever, an empirical intravenous antibiotic therapy with piperacillin/tazobactam and metronidazole was started. Echocardiogram showed normal coronary arteries, minimal pericardial effusion, and mild mitral insufficiency. We were then contacted as reference Center for Kawasaki Disease (KD) in our Pediatric Immunology Unit: considering the clinical history (fever lasting more than 5 days, erythematous rash, labial, and conjunctival hyperemia) and the result of laboratory tests we confirmed the diagnostic suspicion of atypical/incomplete KD without coronary involvement, and started treatment with high dose intravenous immunoglobulins (IVIG) 2 g/kg and high dose acetylsalicylic acid (ASA 50 mg/kg/day). After 36 h from the end of the IVIG infusion the child was apyretic, but abdominal tension with no bowel movements persisted, and mild respiratory distress with tachypnea and oxygen desaturation (91–92%) appeared. Chest X-ray, lung ultrasound, and echocardiogram were performed, showing pulmonary infiltrates at the right base and minimal pericardial effusion. Because of the worsening of respiratory and abdominal conditions, he was transferred to our hospital. When admitted, he was tested for SARS-COV-2 and the nasopharyngeal aspirate resulted positive. Repeated family history revealed that mother and grandmother had been symptomatic for fever, gastroenteritis, and cough in the previous weeks, and that later the father had experienced rhinitis, fever, cough, and ageusia. Because of mild respiratory distress, the patient needed low-flow oxygen administration for 3 days. A repeated chest X-ray showed only accentuated bronchovascular markings in bilateral peri-hilar and paracardiac regions. Considering the persistence of abdominal pain, no passage of stool or gas for more than 48 h from admission, a new abdominal x-ray without contrast was performed, highlighting ileocolic meteorism with multiple small diffuse air-fluid levels. Abdominal pain with distension and lack of bowel movements were compatible with paralytic ileus associated with intestinal vasculopathy in KD. Enema and polyethylene glycol (PEG) oral therapy resolved the abdominal symptoms, ASA 5 mg/kg/die therapy was started after 48 h of apyrexia, and laboratory blood tests showed progressive reduction in inflammation markers and rapid normalization of liver enzymes. In the setting of COVID-19 there are several reports of using corticosteroids for Acute Respiratory Disease Syndrome (ARDS), but considering the not severe clinical course and presentation in our patient we did not start steroid therapy. At discharge echocardiogram showed normal coronary arteries, mild mitral insufficiency, and resolution of pericardial effusion. Nasopharyngeal swab and aspirate for SARS-COV-2 resulted positive, so the family was instructed to keep him in quarantine at home, until repetition of SARS-COV-2 test visit and follow-up visit for KD, which are scheduled in the next future.

## Discussion

KD is an acute, febrile, self-limited systemic vasculitis of small and medium sized vessels and represents the most common cause of acquired heart disease in children in developed countries (6).

Typical or classic KD is characterized by the presence of ≥5 days of fever and ≥4 of the following clinical features: bilateral non-exudative conjunctivitis, erythema of lips and oral mucosa, changes in the extremities, skin rash, and cervical lymphadenopathy with or without coronary artery abnormalities (CAA). Incomplete KD occurs in patients presenting a typical fever without a sufficient number of main clinical criteria, with or without CAA. Atypical KD occurs in patients presenting typical fever and signs or symptoms different from the main clinical features of KD (e.g., meningeal inflammation, seizures, facial paralysis, acute abdomen, acute pancreatitis, cholestatic jaundice, arthritis, renal injury, pneumonia, etc.), with or without CAA (6, 7).

The cause of KD is still unknown but may be related to the combined effects of infections, immune response, and genetic susceptibility. Many studies have reported the role of viral infections in KD as a possible trigger, including enterovirus, rhinovirus, herpesviruses, adenoviruses, influenza, parainfluenza, and coronaviruses (8, 9).

In our case the child had a demonstrated infection by enterovirus, rhinovirus, and SARS-COV-2 according to PCR tests. Many viral infections may elicit systemic inflammation and consequently vasculitides such as KD, and it is also known that SARS-CoV-2 infection can activate uncontrolled inflammation which can lead to local and systemic tissue damage (10). In our patient, more than one pathogen were detected, making it difficult to understand which of the three viral agents had been the trigger.

Our hospital protocol for KD, based on international guidelines (6, 7), recommends the troponin T and BNP dosage only in case of documented myocarditis and/or pericarditis and/or early aneurysms. Since the patient was IVIG responder, electrocardiograms did not reveal signs of ischemia and the echocardiograms performed did not show coronary artery abnormalities or depression of the ejection fraction, we did not measure troponin T or BNP. Moreover, blood tests performed during admission to our hospital, showed a reduction in CRP and normalization of liver enzymes so other investigations would not provide additional information.

Gastrointestinal involvement is not common in KD; the most common gastrointestinal manifestations include vomiting, diarrhea, abdominal pain, hepatomegaly, paralytic ileus, and hydrops of gallbladder (11, 12). In many cases of KD with gastrointestinal involvement, the typical signs and symptoms of KD often occur after the intestinal ones (11–13), and the delayed appearance of typical symptoms can be a cause of delayed therapy, which in turn can lead to a greater probability of cardiac involvement (13, 14). In our case however suggestive symptoms for KD began simultaneously with intestinal symptoms.

Pediatric presentation of SARS-COV-2 infection include completely asymptomatic subjects and patients presenting with mild symptoms like fever, cough, sore throat, vomiting, nausea, and diarrhea (15). Indeed, the clinical course and presentation of COVID-19 in our patient were not severe, even if he transiently needed respiratory support with low-flow oxygen. Considering the mild respiratory presentation, the absence of pathognomonic signs at chest X-ray and the good clinical evolution, we did not start a specific therapy for COVID-19.

To the best of our knowledge, up to now there is only one other published case of KD with concurrent COVID-19, a 6-month-old baby with a complete form of KD (5). However, cases are being informally reported among pediatricians, and recently patients with severe forms including myocarditis and cytokine storm syndromes have been seen in different countries e.g., Italy, France, Netherlands, and UK. An alert has been issued (https://mailchi.mp/05cc3497cda5/alert-possible-sars-cov-2-related-inflammatory-syndrome-in-children?e=2e634f02aa) emphasizing the apparent rise in the number of children presenting with a multisystem inflammatory state requiring intensive care with overlapping features of toxic shock syndrome and atypical Kawasaki. Interestingly, abdominal pain and gastrointestinal symptoms have been a common feature in these patients, such as in our case. Both COVID-19 infection and KD are characterized by cytokine release, and both can be treated with anticytokines (anti-IL-1 in particular) in severe cases. A cytokine storm syndrome, similar to macrophage activation syndrome, can in fact appear in both conditions (16, 17). The connection between viral infections and KD, the analogies between the severe end of the two conditions, with myocarditis having been reported as well, opens new perspectives with regard to etiopathogenesis. A registry of KD cases associated with SARS-COV-2 infection is currently ongoing in Italy, and hopefully results will shed light on this unusual association.

## Data Availability Statement

The raw data supporting the conclusions of this article will be made available by the authors, without undue reservation.

## Ethics Statement

Written informed consent was obtained from next of kin for the publication of any potentially identifiable images or data included in this article.

## Author Contributions

MC wrote the manuscript and designed the topic. RC and RD designed the topic and revised the manuscript. LB revised the manuscript. SG and RP contributed to clinical diagnosis. All authors read and approved the final manuscript and agree to be accountable for the content of the work.

## Conflict of Interest

The authors declare that the research was conducted in the absence of any commercial or financial relationships that could be construed as a potential conflict of interest.
